# Two cases of EBV infection preceding lymphoma

**DOI:** 10.1007/s00432-022-04145-4

**Published:** 2022-06-22

**Authors:** Jonas Saal, Busher Aboudan, Peter Brossart, Annkristin Heine

**Affiliations:** 1 Medical Clinic III for Oncology, Hematology, Immune-Oncology and Rheumatology, University Medical Center Bonn (UKB), Venusberg-Campus 1, 53127 Bonn, Germany; 2 Center for Integrated Oncology, Aachen/Bonn/Cologne/Düsseldorf (CIO-ABCD), Bonn, Germany; 3 Institute of Experimental Oncology, University Medical Center Bonn (UKB), Bonn, Germany; 4 Institute for Pathology, University Medical Center Bonn (UKB), Bonn, Germany

## Abstract

Infection of lymphocytes with the Epstein-Barr virus (EBV) is a well-documented risk factor for developing lymphoma. The incidence of EBV positivity in lymphoma depends on the subtype and can range from 10% in diffuse large B-cell lymphoma (DLBCL) to 100% in endemic Burkitt lymphoma (BL), (Shannon-Lowe and Rickinson, Front Oncol 9:713, 2019). However, in most cases, EBV infection remains unnoticed until diagnosis of lymphoma is made. EBV seropositivity is present in > 90% of the world’s population. Although mostly asymptomatic, in some cases, EBV can cause clinical symptoms, the most common of which are fever, lymphadenopathy and pharyngitis in infectious mononucleosis. Less common presentations include lymphomatoid granulomatosis and mucocutaneous ulcer. Here we report two cases of patients, who were initially diagnosed with localized EBV infection and reactive B-cell proliferation. After B-cell-directed treatment, both patients developed overt lymphoma, in one case classical Hodgkin’s lymphoma (cHL) and in the other case angioimmunoblastic T-cell lymphoma (AITL).

## Case report

### Patient 1

The first patient, a 65-year-old woman, presented to our hospital with an 8-week history of a sore throat which was initially treated with antibiotics. In the course of the disease, hoarseness and dyspnea developed, which was accompanied by cervical lymphadenopathy.

As lymphoma was the likely diagnosis, a biopsy from a sublingual swelling was taken. Histology showed a hyperplastic lingual tonsil with lymphoid proliferation and EBV-positive blasts. No clonal B-cell proliferation was present, T-cells showed a monoclonal rearrangement with a polyclonal background. The findings were interpreted as a reactive state, most probably caused by EBV infection. This diagnosis was confirmed from an additional lymph node biopsy. A bone marrow biopsy showed no signs of lymphoma. The patient finally reported her grandson having had infectious mononucleosis recently. CD4+ T-cells were reduced to 161/μl (lower limit of normal: 450/μl).

Because of significant dyspnea, prednisolone was administered at a dose of 100 mg/d for 5 days which led to a reduction of lymphadenopathy, resolution of the patient’s symptoms improved and discharge from the hospital after 16 days. CD4+ T-cell counts normalized within 6 weeks.

After a three-month period of well-being, the lymphadenopathy progressed and the patient-reported dyspnea again. She was admitted to the hospital and rituximab (500 mg) was started for suspected EBV reactivation. After five courses, the patient reported improvement of the symptoms, although the MRI showed no significant improvement of the cervical lymphadenopathy.

**Fig. 1:**
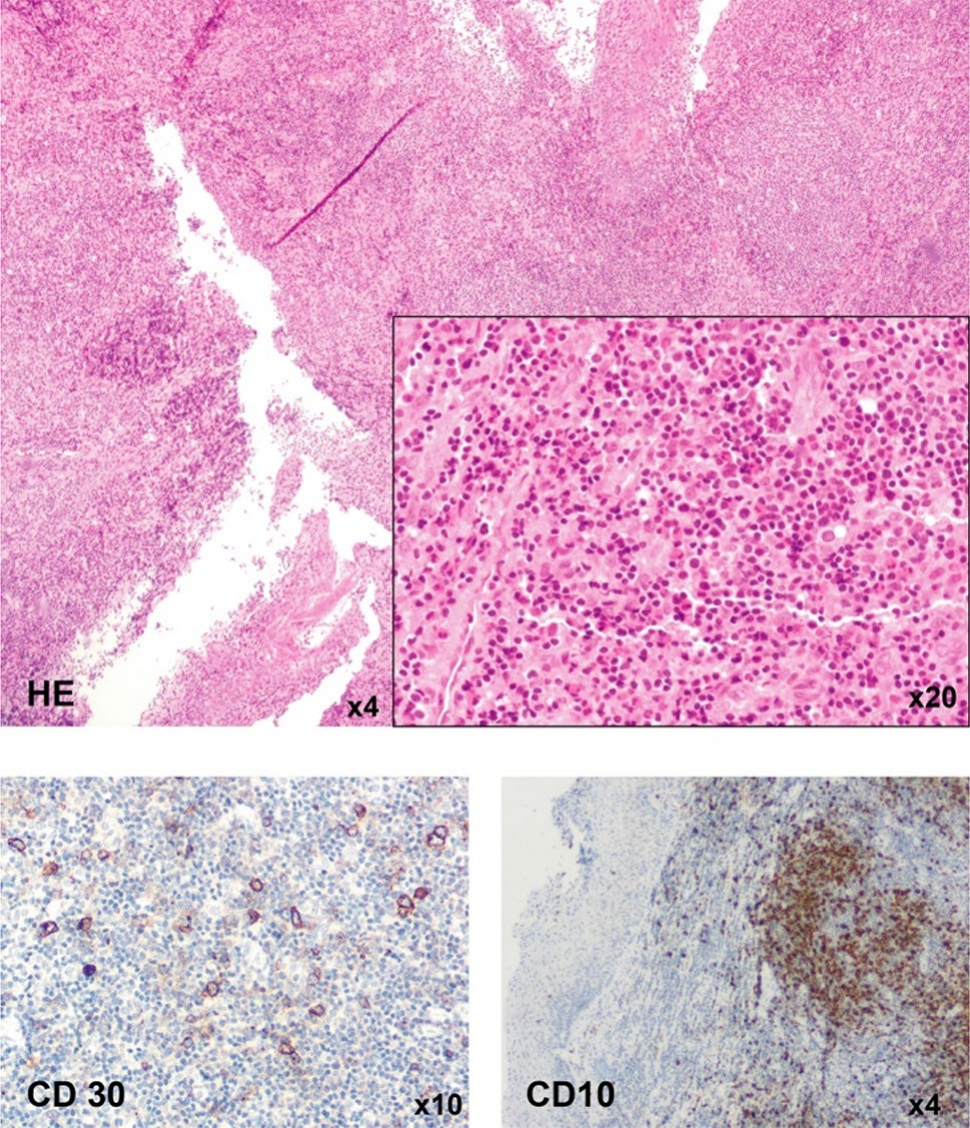
Patient 1 exhibited a lymphoproliferation in a biopsy taken from the sublingual swelling. Partial positivity for CD30 and CD10 was shown in IHC staining. Diagnosis of an EBV-associated lymphoproliferation was made

Six months after the initial presentation, the patient was admitted again with painful cervical swelling. A CT scan showed a mixed response with a marked cervical lymphadenopathy. The decision for a second lymph node biopsy was made. The result was inconclusive, showing a T-cell enriched lymph node without signs of malignancy or EBV positivity (Fig. 1). Another 3 months later the patient was admitted again with progressive dyspnea and cervical swelling. An MRI showed progressive cervical lymphadenopathy. A lymph node was removed surgically and histology now showed an angioimmunoblastic T-cell lymphoma. Retrospectively, AITL could not be diagnosed in any of the prior histology samples. Six cycles of Chemotherapy (CHOEP-21) were administered, followed by high-dose chemotherapy (HD-BEAM) and autologous hematopoietic stem cell transplant (HSCT). Lymphadenopathy resolved completely and the patient improved clinically.

The patient is well, in complete remission without any clinical symptoms, 19 months after auto-HSCT.

### Patient 2

The second patient is a 78-year-old formerly healthy man who presented to our department with a 2-month history of palatal swelling. He had initially presented to a dentist who referred him to maxillofacial surgery. A biopsy showed an inflammatory process. A month later, the patient presented to the ENT department of this hospital with a progressive ulcer on his tongue. An MRI showed cervical lymphadenopathy, which prompted a referral to the department of hematology for suspected lymphoma.

A biopsy of the tongue lesion showed an EBV-associated mucocutaneous ulcer without signs of malignancy. Flow cytometry of peripheral blood showed a monoclonal B-cell population (CD19+ , CD20+ , CD22+ , CD200+ , CD11dim) of approx. 3%. This population was also present in a bone marrow aspirate; histologically there was no sign of lymphoma in the bone marrow. A lymph node biopsy was not performed. CD4+ T-cells were reduced to 118/μl. Rapid growth of the mucocutaneous ulcer prompted the decision to start immunochemotherapy (R-CHOP 21).

**Fig. 2:**
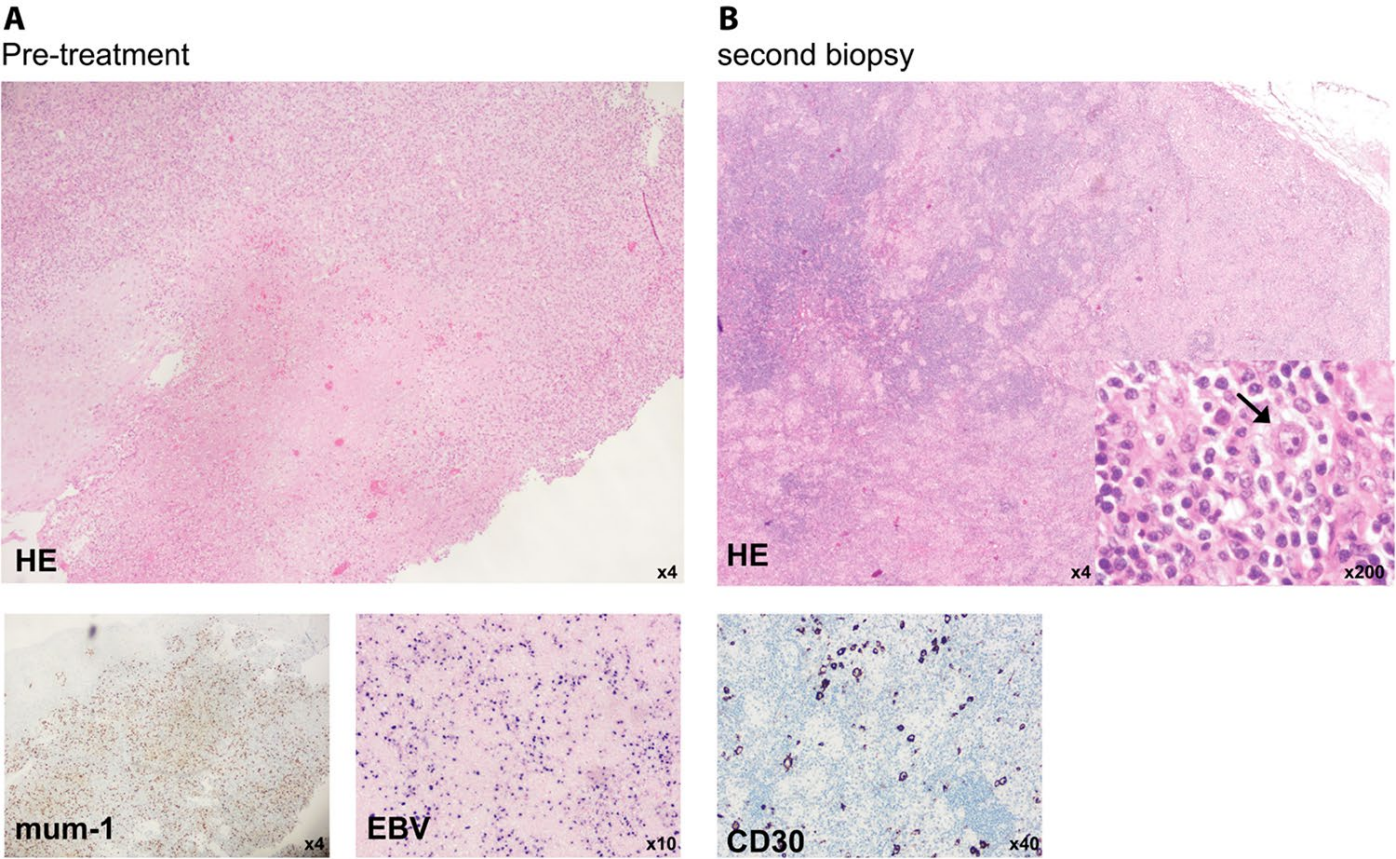
Patient 2 **A** The first biopsy, taken from the tongue lesion, showed a mum-1 positive, mucocutaneous ulcer. Staining for EBV was positive. Diagnosis of an EBV-associated ulcer was made. B The second biopsy, taken after 3 cycles of R-CHOP- followed by Rituximab maintenance treatment, showed typical Reed-Sternberg cells (arrow) in HE-staining. IHC confirmed CD30 expression. Diagnosis of typical Hodgkin-lymphoma was made

The response was mixed, a new submandibular lymphadenopathy developed. A submental mass was biopsied and showed EBV-positive lymphoproliferation. The pathologist ruled out classical Hodgkin’s lymphoma. After 3 cycles of R-CHOP, therapy was deescalated to rituximab (750 mg/m^2^), which was administered every 3 weeks for 8 cycles. The ulcer regressed slowly. Two years and 6 months after initial presentation, the patient presented with a new submental swelling. A biopsy was performed and the diagnosis of classical Hodgkin’s lymphoma was made (Fig. 2). PET-CT scan showed positive cervical and inguinal nodes, resulting in a diagnosis of stage IIIB disease without risk factors according to the Ann-Arbor classification. The patient was started on chemotherapy (AVD) and treated according to the guidelines of advanced stage disease in patients > 60 years.

Both cases initially presented as a benign EBV associated infectious process, however, the aggressive clinical course prompted B-cell directed therapy with rituximab in both cases. This treatment led to an initial improvement of symptoms, indicating a role for EBV-infected B-cells in the patient’s disease. However, the lymphadenopathy progressed after a clinically silent period, leading to repeat biopsies that showed non-B-cell lymphomas. Interestingly, in both patients CD4+ T-cell counts were reduced during EBV infection. While they quickly recovered in patient 1 after steroid treatment, they remained low in patient 2 throughout the entire treatment.

In patient 1, a monoclonal T-cell receptor rearrangement was documented at first diagnosis, but because of a dominant polyclonal background this was considered a reactive process. In patient 2 the suspicion of cHL was raised at first diagnosis, but could not be confirmed pathologically.

Whether rituximab treatment had an effect on lymphoma development by selecting for CD20 negative cells is unclear. However, rituximab treatment did not hinder the development of malignancy in our patients, suggesting that presence of CD20+ B-cells is not necessary for the development of AITL and cHL respectively.

EBV-positive B-cell proliferations are a common finding in AITL, it has been hypothesized that immunosuppression due to T-cell lymphoma facilitates the proliferation of EBV-positive B-cells. EBV in AITL is usually found in accompanying B-cells instead of malignant T-cells. The role of EBV-infected B-cells in AITL is unclear (Zhou et al. 2007). Rituximab has been used for the treatment of AITL; however, a study including 25 patients failed to show a benefit of a combination of rituximab and chemotherapy over chemotherapy alone (Joly et al. 2010).

The development of B-cell lymphoma in the setting of AITL has been described (Willenbrock et al. 2007; Huppmann et al. 2012; Yang et al. 2012).

Despite being different entities, AITL and cHL have striking similarities with regard to tumor composition. In both diseases, malignant cells only constitute a minority of cells present in the malignant lymph nodes. Infiltrating immune cells make up the majority of cells. This facilitates misdiagnoses as reactive processes.

EBV infection is tightly associated with a variety of lymphomas. Our cases emphasize the importance of careful examination and observation of EBV-positive lesions, even if initially appearing benign, as they are at high risk for the development of lymphoma. Thus, inconsistent response to B-cell-directed treatment should raise suspicion of a non-B-cell lymphoproliferative process. In those cases, repeat biopsies should be considered.
